# *In silico* discovery of biomarkers for the accurate and sensitive detection of *Fusarium solani*

**DOI:** 10.3389/fbinf.2022.972529

**Published:** 2022-09-30

**Authors:** Olalekan Olanrewaju Bakare, Arun Gokul, Muhali Olaide Jimoh, Ashwil Klein, Marshall Keyster

**Affiliations:** ^1^ Environmental Biotechnology Laboratory, Department of Biotechnology, University of the Western Cape, Bellville, South Africa; ^2^ Department of Biochemistry, Faculty of Basic Medical Sciences, Olabisi Onabanjo University, Sagamu, Ogun State, Nigeria; ^3^ Department of Plant Sciences, Qwaqwa Campus, University of the Free State, Phuthadithjaba, South Africa; ^4^ Department of Plant Science, Faculty of Sciences, Olabisi Onabanjo University, Ago-Iwoye, Nigeria; ^5^ Plant Omics Laboratory, Department of Biotechnology, University of the Western Cape, Bellville, South Africa

**Keywords:** Fusarium solani, protein, in silico, energies, fungus

## Abstract

*Fusarium solani* is worrisome because it severely threatens the agricultural productivity of certain crops such as tomatoes and peas, causing the general decline, wilting, and root necrosis. It has also been implicated in the infection of the human eye cornea. It is believed that early detection of the fungus could save these crops from the destructive activities of the fungus through early biocontrol measures. Therefore, the present work aimed to build a sensitive model of novel anti-*Fusarium solani* antimicrobial peptides (AMPs) against the fungal cutinase 1 (CUT1) protein for early, sensitive and accurate detection. *Fusarium solani* CUT1 receptor protein 2D secondary structure, model validation, and functional motifs were predicted. Subsequently, anti-*Fusarium solani* AMPs were retrieved, and the HMMER *in silico* algorithm was used to construct a model of the AMPs. After their structure predictions, the interaction analysis was analyzed for the *Fusarium solani* CUT1 protein and the generated AMPs. The putative anti-*Fusarium solani* AMPs bound the CUT1 protein very tightly, with OOB4 having the highest binding energy potential for HDock. The pyDockWeb generated high electrostatic, desolvation, and low van der Waals energies for all the AMPs against CUT1 protein, with OOB1 having the most significant interaction. The results suggested the utilization of AMPs for the timely intervention, control, and management of these crops, as mentioned earlier, to improve their agricultural productivity and reduce their economic loss and the use of HMMER for constructing models for disease detection.

## Introduction

*Fusarium solani* severely threatens agricultural productivity worldwide due to reducing plant crops’ nutrients and harvest, resulting in tremendous economic losses (Hartman et al., 2011). It causes root rots of its host by penetrating plant cell walls and destroying the torus (Raaijmakers et al., 2009). *Fusarium solani* is a common soil fungus of a complex of more than twenty-six closely related filamentous fungi in the Nectriaceae family (Mavhunga, 2020). It is found in ponds, rivers, sewage facilities, water pipes, larvae and adults of the picnic beetle, and a symbiote of the ambrosia beetle (Šišić et al., 2018). It infects plants through developing plant roots to produce asexual macro- and microconidia dispersed through wind and rain (Shakeel et al., 2020). Morphologically, *Fusarium solani* is unique because, unlike most Fusarium species that form a pink or violet centre when cultured, it forms white and cottony colonies with a blue-green or bluish brown colour (Chehri et al., 2015). It is a common cause of diseases in plants such as peas, beans, potatoes, olive, soybeans, and many types of cucurbits and humans, resulting in either mycoses or the infection of the eye cornea. It can result in plant decline, wilting, and necrosis in plant roots (Kurt et al., 2020).

Several researchers have carried out work that focuses on the measures to reduce the menace of *Fusarium solani* on animals and crop plants (Bhat et al., 2016; Mahawar et al., 2019). One such work identified *Fusarium solani* to contain 5–17 chromosomes with a genome size of 45.81 Mbp and above (Coleman et al., 2009). Another research also identified the GC contents of its DNA to be 50% (Rasmey et al., 2020). The mycelium of *Fusarium solani* is rich in alanine and several fatty acids such as δ-aminobutyric-, palmitic-, oleic-, and linolenic acids (Nidiry et al., 2011). Potassium is necessary for the growth of *Fusarium solani*, and when the potassium level reduces to 3mM, it develops a feathery pattern (Mecteau et al., 2008). *Fusarium solani* can decompose cellulose at an optimal pH of 6.5 and a temperature of 30°C (Scully et al., 2012). It can metabolize steroids and lignin and reduce Fe^3+^ to Fe^2+^ (Hurst, 1995; Xiang et al., 2021). This fungus also produces several toxins such as mycotoxins (trichothecenes and fumonisins), and other toxins produced from citrus-associated F. solani include napthozarins, while certain toxic metabolites such as solaniol, neosolaniol, T-2 toxin, HT-2 toxin, and diacetoxyscirpenol (Rehman et al., 2012).

*Fusarium solani* is a stubborn plant pathogen because it is unaffected by the pH changes of the soil significantly, and soil fumigation increases its occurrence (Bhatti et al., 2013). This tendency allows *Fusarium solani* to persist in the soil for at least a decade to wreck its complete crop loss. Its virulence in plants is partly controlled by the cutinase 1 (CUT1) gene, upregulated by exposure to the plant’s cutin monomers (Li et al., 2002). A plethora of management practices exist which are developed independently due to the ubiquitous nature of the fungus. Despite these management practices, the menace of this fungus on plant crops is becoming alarming (Agnoli et al., 2012). It is of significant note to describe the functional and structural architecture of homologous cutinase 1 (CUT1) since its gene controls the virulence of the fungus. The circular dichroism and fluorescence profile at different pH ranges of 6–9 showed unique structural formation for the cutinase, indicating its stability to a wide range of pH (Pham et al., 2016). There is a high resemblance of the secondary structure of the cutinase using homology modelling for its structural study across *Fusarium solani* (Wei et al., 2014). However, the structural stability of the cutinase differs significantly in its tertiary structure, hydrophobicity, electrostatic parameters, and across different tolerance levels in folding during denaturation to aqueous guanidine hydrochloride. The four tryptophan residues in the protein is embedded in the inaccessible hydrophobic pockets. There is a different distribution of the aromatic amino acid on the surface of the enzyme (Kruithof, 2007).

Several methods exist for diagnosing *Fusarium solani* in the laboratories for its sensitive and timely detection. One of them relies on clinical observations such as hyaline hyphae in tissue, necrotic lesions in the skin and positive blood tests with fungal growth or the presence of fungal cell wall components to hint at fusariosis (van Diepeningen et al., 2015). Several laboratories also rely on morphological identification. However, multi-locus sequencing discriminates among species complex members (Zaccardelli et al., 2008). Diagnostic tools based on DNA identification have the best discriminatory power when based on translation elongation factor 1-α or the RNA polymerase II second largest subunit (He et al., 2011). The use of antimicrobial peptides could be used for disease diagnostics when modelled using powerful in silico tools such as HMMER (Tincho et al., 2016; Williams and Tincho, 2016; Bakare et al., 2020; Bakare et al., 2021a). Despite these interventions, the rapid test for the fungus has been suggested by authors for timely detection before it wreaks its havoc on the host plant.

Antimicrobial peptides (AMPs) are small peptides that exist in nature and are part of many organisms’ innate systems with tested inhibition against bacteria, viruses, fungi, and other parasites (Bahar and Ren, 2013). The incidence of antibiotic-resistant microorganisms and the knowledge of the therapeutic effects have been well-described. It was discovered only recently that the wide-ranging functionality of the AMPs against diseases and infections expands the list of activities beyond antimicrobial effects attributed to them (Zhang and Gallo, 2016). AMPs have found applications in diagnostics where they are said to have a wide range of activities against HIV, bacterial and viral pneumonia, and *Fusarium oxysporum* (Tincho et al., 2016; Williams and Tincho, 2016; Bakare et al., 2020; Bakare et al., 2021a; Bakare et al., 2021b). Therefore, this work aimed to use *in silico* algorithms such as HMMER to build antimicrobial peptide models against *Fusarium solani* cutinase 1 for sensitive identification. This is important for its early detection for timely intervention, control, and management to improve agricultural productivity of crop plants such as peas, beans, potatoes, olive, soybeans, and many types of cucurbits and mycoses in humans.

## Materials and methods

### Retrieval of receptors

The gene for the receptor, CUT1 protein, was identified for *Fusarium solani* (isolate Cutin hydrolase 1; Flags: Precursor) and collected from the National Center for Biotechnology Information (NCBI) (https://www.ncbi.nlm.nih.gov/, accessed on 26 December 2021) (Pruitt et al., 2005), through literature mining. Thereafter, verification was performed using curation to ensure that the retrieved *Fusarium solani* gene was complete and specific for *Fusarium solani*. Translation of the reading frame of the coding portion of the gene into protein was performed using the Ex-PAsy translate tool (https://web.expasy.org/translate/, accessed on 27 December 2021) (Artimo et al., 2012). BLAST analysis was then performed using the UniProt interface (https://www.uniprot.org/help/uniprotkb, accessed on 23 January 2021) for further assurance of specificity such that the CUT1 protein of interest was specific for *Fusarium solani*.

### 2-D secondary structure prediction

The 2-D secondary structure prediction of the CUT1 protein was carried out using the PSIPRED server to ascertain their alpha-helices, beta-sheets, and random coils (http://bioinf.cs.ucl.ac.uk/psipred, accessed 30 December 2021) (McGuffin et al., 2000).

### Protein model evaluation

The quality of the resulting CUT1 protein model was checked using PROCHECK (https://www.ebi.ac.uk/thornton-srv/software/PROCHECK/, accessed 31 December 2021) to predict parameters such as chain length, hydrogen bond geometry, planarity and angles of the peptide bonds (Laskowski et al., 2006).

### Prediction of functional motifs

The MOTIF finder (http://www.genome.jp/tools/motif/, accessed 30 January 2022) was used to find the motifs present in the protein to enable its complete description (Carlson et al., 2007).

### Anti-*Fusarium solani* AMPs collection

Collection of anti-*Fusarium solani* AMPs was carried out from antimicrobial peptide databases such as Antimicrobial Peptides Database (APD3) (https://aps.unmc.edu/database/anti) (Wang and Wang, 2004; Wang et al., 2005) and Collection of Antimicrobial Peptides (CAMP) (http://www.camp.bicnirrh.res.in/seqDb.php?page=0) (Thomas et al., 2010). Thereafter, the confirmation that the collected AMPs were either experimentally validated or predicted mining was carried out using literature mining. Removal of duplicate experimentally validated AMPs was then ensured from the list using the Cluster Database at High Identity with Tolerance (CD-HIT) (Li and Godzik, 2006).

### Data partitioning

Random partitioning into two subsets (3/4 of the data utilized as the training partition and the remaining ¼ utilized as testing) of the screened experimentally validated AMPs was carried out to build a strong profile, including optimization/calibration of profiles.

### Construction of profiles

Utilization of the Hidden Markov Models (HMMER) algorithm version 3.8 (Roddy, 2018) was carried out to build pathogen-specific profiles using the constructed datasets utilizing the terminal of the Ubuntu operating system version 12.04; (Canonical Ltd., London, United Kingdom) with the command line used for building the profile written:

For the first step, the training datasets were aligned using the Clustalo alignment tool (Fang, 2018). The alignment was carried out using the command line:

Clustalo –i FSTrainings. Fasta –o FSTrainings.sto --outfmt=st (i)

The command line simply states <<do an alignment of the sequences which are in the upper case found in the input file “dataset.fasta” with the Fasta, using Clustalo as multiple alignment tools and GCG Postscript output for graphical printing>>. The output of the command results in the construction of aligned sequences called “dataset.msf.” The aligned sequences were used as input in the next step.

The next step enhances the construction of the profiles of the target class sequences by showing the common motifs/signatures within the profiles. To achieve this, the “Build profiles” was run using the following command:

hmmbuild FSTrainings.hmm FSTrainings.sto (ii)

**FIGURE 1:**
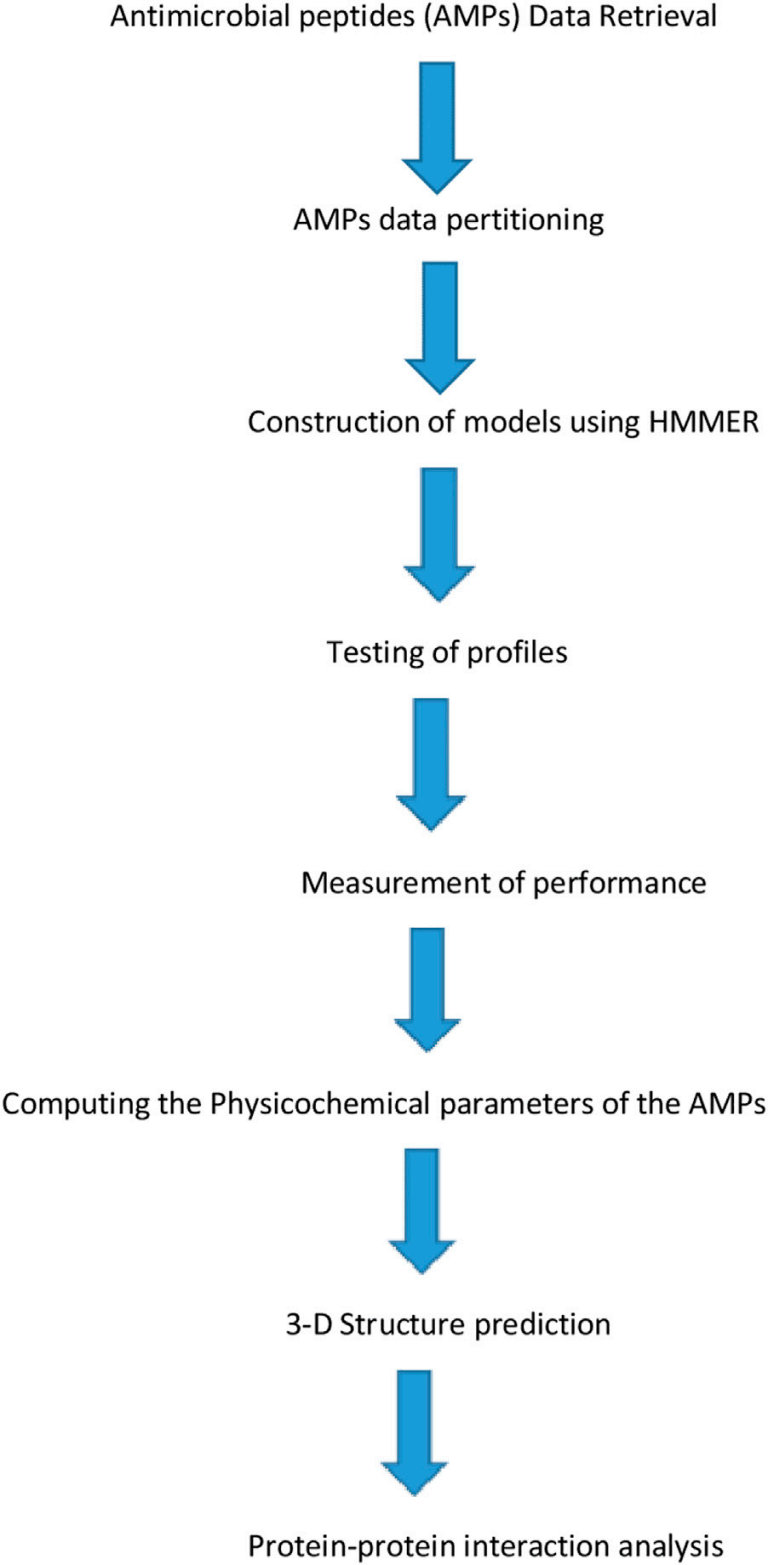
Detailed representative structural representation for Antimicrobial peptides (AMPs) model construction.

The resulting profiles “dataset.hmm” was used in evaluating the profiles’ performance by testing the created profiles on an independent AMP dataset. Figure 1 below shows the detailed representation of AMPs model construction.

### Testing of profile

The query of the profile was carried out in a step called “Query profiles” utilizing the testing data against that profile using the command line as follows:

The independent testing of each created profile was performed in a step called “Query profiles.” The testing data were queried against the created profiles using the command line, with an E-value threshold of 95% or 0.05:

Hmmsearch –E5e-2 FSTrainings.hmm profile query.txt > resultfile.txt (iv)

### Measurement of performance of the profile

Sensitivity, specificity, accuracy, and Matthews Correlation Coefficient as statistical parameters were carried out as described below, where TP indicates true positive, TN indicates true negative, FP indicates false positive, and FN indicates false negative:

Percentage sensitivity of the anti-*Fusarium solani* AMPs against *Fusarium solani* (testing sets) effectively predicted as anti- *Fusarium solani* AMPs (positive). The equation of the sensitivity is written below as (1):

$$\text{Sensitivity}=\left(\frac{TP}{TP+FN}\right)\times 100$$ (1)

Percentage specificity of the non-anti- *Fusarium solani* AMPs (negative sets) effectively predicted as non-anti- *Fusarium solani* AMPs (negative). The equation of the specificity is written below as (2):

$$\text{Specificity }=\left(\frac{TN}{TN+FP}\right)\times 100$$ (2)

Percentage accuracy of the effectively predicted peptides (anti- *Fusarium solani* AMPs and non-anti- *Fusarium solani* AMPs). The equation of the accuracy is written below as (3):

$$\text{Accuracy }=\left(\frac{TP+TN}{TP+FP+TN+FN}\right)\times 100$$ (3)

Matthew’s correlation coefficient (MCC) measures the sensitivity and specificity. MCC = 0 is an indication of absolutely random prediction, while MCC = 1 means perfect prediction. *See* the Eq. 4 as below:

$$\text{MCC }=(\frac{\left(TP\times TN)-(FN\times FP\right)}{\sqrt{(TP+FN)\times (TN+FP)\times (TP+FP)\times (TN+FN)}})$$ (4)

### Identification of putative anti- *Fusarium solani* AMPs

Proteome sequences were scanned using the profile with the list of all proteome sequences retrieved from the Ensembl database (http://www.ensembl.org/index.html, accessed on 22 April 2021) (Hubbard et al., 2002) and the UniProt database (http://www.uniprot.org/, accessed on 23 April 2021) (Consortium, 2015). An E-value cut-off was set to 0.05 for the discovery of putative anti- *Fusarium solani* AMPs. The task was accomplished using “hmmsearch” module of the HMMER software with the command line employed stated as follows:

Hmmsearch –E5e-2 FSTrainings.hmm profile query.txt > resultfile.txt (iv)

Specific FSTrainings.hmm in the profile, target class query.txt representing the species scanned against the profile and resultfile.txt is the output file acquired after testing the species against the constructed *Fusarium solani* (FS) profile.

### Computation of the physicochemical properties of the putative anti- *Fusarium solani* AMPs and the *Fusarium solani* CUT1 protein

The anti- *Fusarium solani* AMPs physicochemical properties were calculated using the prediction interface of BACTIBASE (http://bactibase.pfba-lab-tun.org/physicochem, accessed on 31 June 2021) (Hammami et al., 2007; Hammami et al., 2010), DBAASP (https://dbaasp.org/, accessed on 31 July 2021) (Pirtskhalava et al., 2016), and APD3 (https://wangapd3.com/main.php, accessed on 28 August 2021) (Wang et al., 2009) and the receptor CUT1 protein was carried out using ProtParam tool (http://web.expasy.org/protparam/, accessed on 28 August 2021) from the ExPAsy server (Kyte and Doolittle, 1982).

### Predictions of the putative anti- *Fusarium solani* AMPs and *Fusarium solani* protein structures

An example of a *de novo* peptide or protein structure prediction method was used to generate the putative anti- *Fusarium solani* AMPs, and the *Fusarium solani* CUT1 protein structures such as I-TASSER (Iterative Threading ASSembly Refinement) server was utilized (Eswar et al., 2009). In brief, uploading of each sequence onto the I-TASSER website was performed (Roy et al., 2010), and RasMol 2.7.5 Software (NextMove Software Ltd., Cambridge Science Park, United Kingdom) was then utilized to visualize the 3-D structures of the AMPs and the protein receptor (Sayle and Milner-White, 1995).

### Putative anti- *Fusarium solani* AMPs and *Fusarium solani* protein interaction analysis

The pyDockWeb web-server which allows the docking of the protein-small ligand molecule, available at https://life.bsc.es/servlet/pydock/ (accessed on 31 March 2022) was used for the docking of the anti- *Fusarium solani* AMPs to the *Fusarium solani* CUT1 protein (Jiménez-García et al., 2013). In brief, the I-TASSER-generated PDB files for the 3-D structures of the anti- *Fusarium solani* putative AMPs and the *Fusarium solani* protein receptor were uploaded onto the pyDockWeb server. The interaction analysis of the complex between the anti- *Fusarium solani* putative AMPs and the CUT1 protein receptor was achieved using RasMol 2.7.5 Software (NextMove Software Ltd., Cambridge Science Park, United Kingdom) (Sayle and Milner-White, 1995). Subsequently, binding scores of the complex formed between the AMPs and the receptor protein were computed using the HDock server (http://hdock.phys.hust.edu.cn/, accessed on 3 March 2021) for comparison (Yan et al., 2020).

## Results

### 2-D model structure prediction

The predicted result represented by PSIPRED server from Supplemetary1 revealed that the secondary structure of CUT1 protein contained 100 small non-polar, 46 hydrophobic, 61 polar, and 19 aromatics plus cysteine regions necessary to strengthen its activity.

The predicted result represented by the PSIPRED server further revealed that the secondary structure of CUT1 protein contained 6 beta-strands, 10 alpha-helices, and 16 random coils (Supplementary 2). The structure revealed that CUT1 protein belongs to an alpha-beta class, with a central beta-sheet of 4. The abundance of alpha-helices allowed the protein to perform its functions to act on carboxylic ester bonds and facilitate the fungus penetration into the plant cuticle.

### 3-D modelled structure validation

The structural quality of the modelled CUT1 was carried out using PROCHECK (https://servicesn.mbi.ucla.edu/ PROCHECK/). As indicated in Supplementary 3, the PROCHECK result analysis indicated that CUT1 had 92.6% residues in the most favored regions, 6.8% in the additional allowed regions, and 0.6% in the generously allowed and 0.0% at the disallowed regions. The distribution of the amino acid residues made its model of high quality.

### Prediction of motif regions

Table 1 shows the probable functional motifs of CUT1 protein with three motifs in which the first one was located at position 46.222 in the amino acid sequence with an E value of 7e–53. Vir1 was located at position 118.171 in the amino acid sequence with an E value of 0.035, and the Mbeg1-like motif was located at position 120.16 in the amino acid sequence with an E value of 0.11.

**TABLE 1:** Motif regions analysis.

Pfam ID	Pfam ID number	Position	Independent E value	Description
Cutinase	PF01083	46.222	7e-53	Cutinase
Vir1	PF06057	118.171	0.035	Bacterial virulence protein
Mbeg1-like	PF11187	120.16	0.11	Mbeg1-like

**FIGURE 2:**
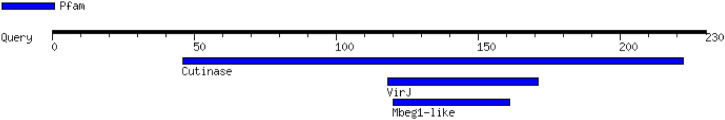
Promising functional motifs present in CUT1 protein predicted by Motif finder (Number of motifs is 3, and it is virulence protein).

The motif prediction of CUT1 protein *Fusarium solani* revealed that the fungus contains three small regions of the three-dimensional protein structure or amino acid sequence known as motifs which had virulence hydrolase, and (Figure 2).

### Antimicrobial peptides (AMPs) data collection and profile construction

Profile creation was carried out by random division of the experimentally validated anti-*Fusarium solani* antimicrobial peptides (AMPs) (Table 2). Subsequently, HMMER was used to cluster, build, and scan putative AMPs with the tendency to detect *Fusarium solani.* The experimentally validated anti-*Fusarium solani* AMPs were collected from CAMP, APD3, DBAASP, and BACTIBASE in which literature mining revealed the presence of 16 AMPs against *Fusarium solani* after removal of duplicate.

**TABLE 2:** Profile creation partitioning.

S/N	Profile	Training dataset	Positive dataset	Negative dataset
1	FS	12	4	17236

### Testing of performance of the profile

The created profile of the anti-Fusarium solani AMPs was tested against the positive dataset, which was 25% of the total AMPs used to create the profile. A negative control dataset was also used, containing a random fragment of 17236 neuropeptides with no anti-*Fusarium solani* activities (Table 3). The result revealed that only three of the four positive testing datasets were true positive while the profile discriminated against all the 17236 negative datasets (neuropeptides). The performance results also revealed that the profile was sensitive, accurate, and specific, with much significant Matthews correlation coefficient (MCC) (Table 2).

**TABLE 3:** Independent testing of the profile.

S/N	True positive	False negative	True negative	False positive
1	3	1	17236	0
S/N	Sensitivity (%)	Specificity (%)	Accuracy (%)	MCC
1	75	100	99.99	0.87

MCC, Matthews correlation coefficient.

### Discovery of anti-*Fusarium solani* AMPs

Novel anti-*Fusarium solani* AMPs were discovered using HMMER with a set cut-off E value at 0.05. This yielded five AMPs across all proteomes scanned which adhered to the cut-off (Table 4). The AMPs were ranked according to their E values, with the lowest coming first on the list.

**TABLE 4:** Discovery of anti-*Fusarium solani* AMPs.

S/N	AMP name	Anti-Fusarium solani AMPs	Organism scanned	E value	Bit scores
1	OOB1	NTQEIIKRTCSGNSEKSKGRILPTERTPISRRKTEQPLVGQCEAHLVMGQCLCQSLCDSQDQQHKSTAKSSLVIDAGCSEIGT	*Ictidomys tridecemlineatus*	1.3e−08	21.5
2	OOB2	TPNQRQNVCAENEGIPDGACSKDSDCHAGEAVTAGNGVKTGRCLRRENLARGTCE	*Homo sapiens*	6.8e−07	16.0
3	OOB3	DGKHSGKNLSNAQFGRGGCTEECVSAKRNVPTSALYQVCTRTLKSTGQGQTSFSSLTPCTAK	*Anolis carolinensis*	4.3e−07	16.6
4	OOB4	RLTAKCLCSRPARRRRGCHLARWPLPLCADEL	*Dasypus novemcinctus*	2.6e−07	17.3
5	OOB5	TPNQRQNVCAENEDIPDGACSEDSDCHSGEAVTAGNGVKTGRCLWRENLTRGTCE	*Callithrix jacchus*	1.7e−06	14.7

OOB1-5, anti-*Fusarium solani* AMPs.

### Physicochemical analysis of the anti-*Fusarium solani* AMPs and *Fusarium solani* CUT1 protein

Physicochemical parameters such as molecular weight, isoelectric point, percentage hydrophobicity, Boman index, net charge, and half-life were used to evaluate the resulting anti-*Fusarium solani* AMPs (Table 5). OOB1-5 had their most common amino acids: serine, glycine, threonine plus serine, arginine, and glutamate plus glycine. All the AMPs had significant hydrophobicity, with the lowest recorded for OOB3 (27%) and 5 (29%), which revealed the total percentage hydrophobicity as recorded for both APD3 and BACTIBASE. All the AMPs had positive charges with the exception of OOB2 and 5, which had 0 and −4, respectively. The isoelectric point of the AMPs was between 4.23 and 11.17, with the Boman index ranging from 2.14 to 2.99. Also, all the AMPs had a significant half-life, with the lowest recorded for OOB4 (1 h).

**TABLE 5:** Physicochemical properties of the anti-*Fusarium solani* AMPs.

S/N	AMP	Molecular weight (Da)	Common amino acid	pI	% Hydrophobicity	Boman index (Kcal/Mol)	Net charge	Half-life (hours)
1	OOB1	9048.93	S	8.13	30	2.42	+2	1.4
2	OOB2	5762.14	G	5.74	31	2.93	0	7.2
3	OOB3	6497.67	TS	9.52	27	2.14	+6	1.1
4	OOB4	3719.68	R	11.17	47	2.84	+7	1
5	OOB5	5897.15	EG	4.23	29	2.99	−4	7.2

pI, Isoelectric point.

In Table 6 below, CUT1 protein of *Fusarium solani* had alanine as the most common amino acid, molecular weight of 23985.36 Da, 52% hydrophobicity, the isoelectric point of 8.13, net charge of +3 and a half=life of 30 h. The protein also had instability and aliphatic indices of 22.84 and 87.17, respectively.

**TABLE 6:** Physicochemical properties of the *Fusarium solani* CUT1 protein.

S/N	Molecular weight (Da)	Common amino acid	pI	% Hydrophobicity	Instability index	Net charge	Half life (hours)	Aliphatic index
1	23985.36	A	8.31	52	22.84	+3	30	87.17

pI, Isoelectric point.

### Structure prediction of the anti-*Fusarium solani* AMPs and Fusarium solani CUT1 protein

The structures of the anti-*Fusarium solani* AMPs and *Fusarium solani* CUT1 protein were predicted utilizing parameters such as confidence score (C-score), Template modelling score (TM score), and Root means square deviation (RMSD) (Å) (Table 7). C-score is used to estimate the quality of the prediction by I-TASSER based on the significance of threading template alignments and the convergence parameters of the structure assembly simulations. C-score ranges from −5 to 2 for a model with high confidence (Zhang, 2008). All predicted models had significant C-score indicating that the 3-D structures of the putative AMPs and CUT1 protein were predicted with high confidence. Also, a TM score >0.5 indicates a model of correct topology and a TM-score < 0.17 means a random similarity. OOB2, 5, and CUT1 had correct topology, while OOB1, 3, and four had random similarities. The random similarity could be due to a lack of templates for predicting these molecules, an indication of their novelty (Bakare et al., 2020). For RMSD, 3-D structure prediction does not have a definitive RMSD value (Bakare et al., 2021a).

**TABLE 7:** Structure prediction of the anti-*Fusarium solani* AMPs and *Fusarium solani* CUT1 protein from I-TASSER.

S/N	ITASSER Code	AMPs	C-scores	TM scores	RMSD (Å)
1	S670897	OOB1	−3.60	0.32 ± 0.11	11.6 ± 4.5
2	S672774	OOB2	0.06	0.72 ± 0.11	2.7 ± 2.0
3	S675961	OOB3	−2.80	0.39 ± 0.13	8.9 ± 4.6
4	S676363	OOB4	−2.32	0.44 ± 0.14	6.4 ± 3.9
5	S676629	OOB5	−0.17	0.69 ± 0.12	3.1 ± 2.2
6	S675713	CUT1 protein	0.38	0.76 ± 0.10	4.8 ± 3.2

C-scores, Confidence scores; TM, Template Modelling scores; RMSD, Root mean square of the Deviation.

**FIGURE 3:**
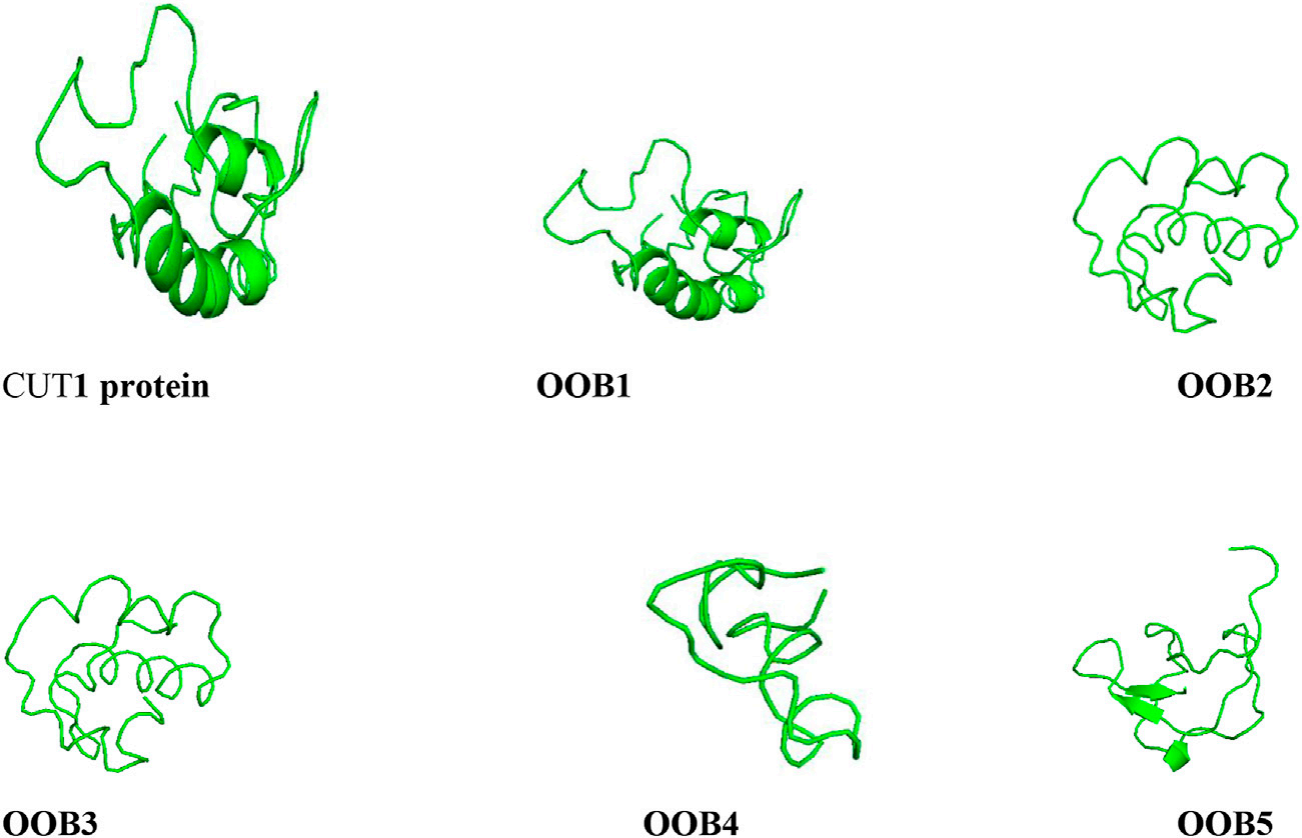
Structures of the anti-*Fusarium solani* AMPs and *Fusarium solani* cut1 protein all represented in ash colour visualized using PyMol.

The output images of the anti-*Fusarium solani* AMPs from the I-TASSER server and the CUT1 receptor are displayed in Figure 3. The representative 3-D structures showed that the putative AMPs and CUT1 protein displayed various secondary structures, α-helices, parallel β-sheet, anti-parallel β-sheet, extended, and loop conformational structures (Ramamoorthy et al., 2006; Sengupta et al., 2008).

### Docking interaction analysis of the anti-*Fusarium solani* AMPs and *Fusarium solani* CUT1 protein

The docking interaction analysis between the anti-*Fusarium solani* AMPs and *Fusarium solani* CUT1 protein was predicted using pyDockWeb and HDock servers (Table 8). All the anti-*Fusarium solani* AMPs bound tightly with the CUT1 protein with the highest binding energy displayed for OOB4 in HDock. Despite high electrostatic, desolvation, and low van der Waals interaction energies generated for all-AMPs by pyDockWeb, OOB1 was ranked most significant.

**TABLE 8:** Docking interaction analysis of the putative AMPs and CUT1 protein using pyDockWeb and HDock.

S/N	AMPs	pyDockWeb electrostatic energy	pyDockWeb desolvation energy	PyDockWeb van der Waals forces	HDock
1	OOB1	−24.637	−7.934	29.582	−186.70
2	OOB2	−4.500	−22.345	41.744	−191.13
3	OOB3	−4.500	−22.345	41.744	−191.96
4	OOB4	−8.074	−21.295	53.338	−203.77
5	OOB5	−17.615	−30.273	67.820	−178.16

**FIGURE 4:**
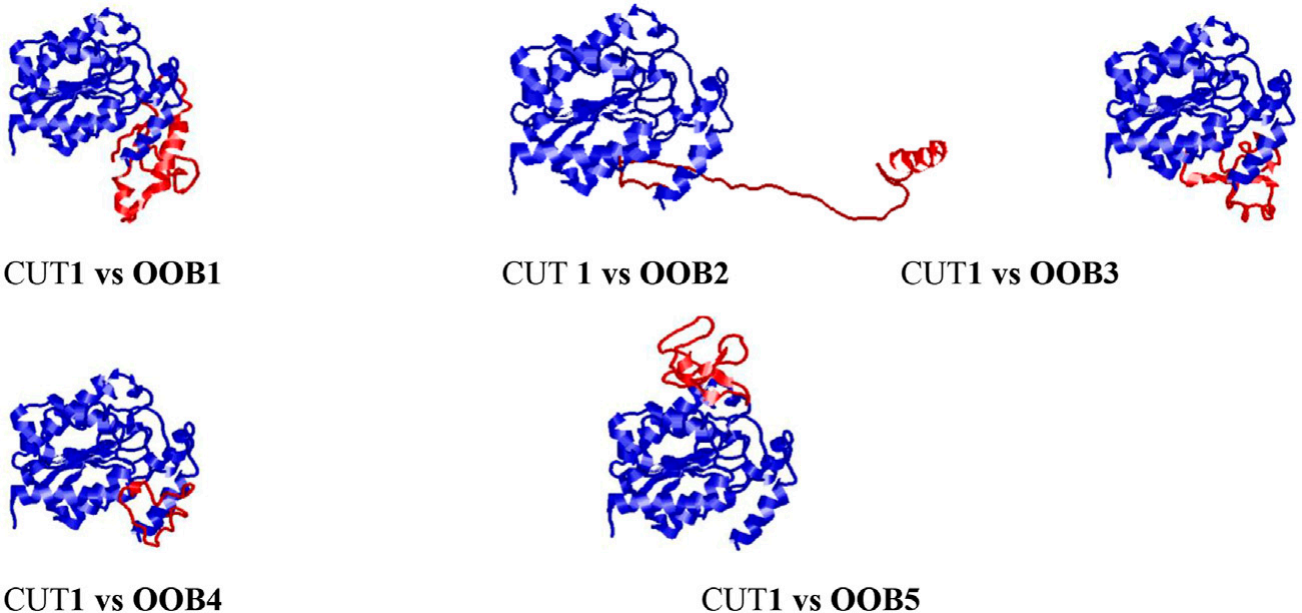
Docking interaction complexes between cut1 receptor protein (represented in blue) and anti-*Fusarium solani* AMPs (represented in red) visualized using RasMol.

The output images from the HDock server showing the mode of binding and orientation of the anti-*Fusarium solani* AMPs along the *Fusarium solani* CUT1 protein are displayed in Figure 4. OOB1, 2, 3, and four had similar binding modes and orientations along the CUT1 protein, with only OOB5 binding differently. The observed differences in the orientation could be attributed to the interaction between the amino acid residues of the anti-*Fusarium solani* antimicrobial peptides and the CUT1 respectively (Jaakola et al., 2010).

## Discussion

Identification of *Fusarium solani* through model construction of detection biomarkers could pave the way toward saving infected crop plants for an abundant harvest and greater economic value. The present research identified CUT1 protein as a target for detecting *Fusarium solani* during infection. CUT1 protein of *Fusarium solani*, as used in this research, is a stable protein based on its fortification with polar, non-polar, aromatic, and cysteine amino acids, which were contributed by the hydrophobic core, hydrogen bonding, net charge and the ionization state of the amino acid residues, a necessary criterion for receptor molecule used for identification purposes (Pace et al., 2014). This receptor protein had an excellent and unique model structure prediction in its secondary structure. The presence of chemical forces between protein and its immediate environment of CUT1 and the noncovalent bonds between amino acids could also explain its stability (Pace et al., 2014). The disulphide bridges in this receptor molecule were essential for protein stability, in which their disruption could result in loss of enzymatic activity (Pace et al., 2009).

The *Fusarium solani* CUT1 also displayed a high quality in its model validation with abundant most favoured regions for the reception of ligands capable of being biomarkers. Also, the three motifs of the fungal CUT1 protein are recognizable regions of protein structure with unique virulence functions for penetration into the host plant cuticle (Hanschen et al., 2014). The essence of the validation and motif finding steps was to ascertain that the CUT1 protein had evolutionarily more conserved regions for *Fusarium solani* than other regions of proteins in other organisms (Rost et al., 2003). Identifying motifs in proteins is necessary for classifying protein sequences and detecting functional annotation (Wu et al., 2003). Thus, CUT1 protein of *Fusarium solani* had promising functional motifs ranging from hydrolytic to virulent functions apart from being an evolutionarily conserved molecule.

AMPs have gained widespread attention from researchers as theranostic molecules because of their compensatory advantages over conventional antibodies during diagnosis and ease of penetration due to their favourable biochemical nature and small size (Tincho et al., 2016; Aruleba et al., 2018). The use of HMMER for the construction of models to identify pathogens as used in this research work is deemed appropriate in the field of diagnostics due to its correct prediction of models against specific organism types (Tincho et al., 2016). A model of the retrieved AMPs was constructed using HMMER after random partition into two datasets. The essence of the arbitrary partition exercise of the datasets into training and testing and the independent testing of the profile was to ascertain the robustness and the discriminatory power of the profile built by HMMER (Wu et al., 2003). The anti-Fusarium solani AMP model constructed was sensitive, accurate, and specific, with an excellent Matthews correlation coefficient using performance parameters. The model generated five anti-Fusarium solani AMPs across all proteomes of organisms scanned with significant E-values less than 0.05, with the lowest E-value recorded for 00B1.

The physicochemical parameters of the AMPs were computed to ascertain that they were *bona vide* AMPs. The Boman index was greater than 1 for all AMPs, indicating the greater capacity to bind to its receptor during pathogen detection with their percentage hydrophobicity above 30% except 00B3 and 5. The reduced hydrophobicity of OOB3 and 5 is the proportion of the polar amino acids above the non-polar ones. All the AMPs generated were cationic except OOB2 with a neutral charge. The absence of a positive charge does not interpret the absence of antimicrobial activity because some non-cationic AMPs have been reported with more excellent antimicrobial activities (Bakare et al., 2020).

The pyDockWeb generated three energy interactions: electrostatic, desolvation, and van der Waals interaction energies (Jiménez-García et al., 2013). The different structural formations such as alpha-helices and extended sheets of the putative anti-Fusarium solani AMPs in their 3-D structures can help folding complementation, insertion and intermolecular interaction (Aruleba et al., 2018). This is because alpha-helices exhibit efficient use of hydrogen bonds during the binding of the amino group hydrogen and the carboxyl group oxygen. Thus, the presence of alpha-helices in the AMPs makes them interact with another biomolecule for significant impact as targets during detection (Kumar et al., 2018). The 3-D structure of the CUT1 protein and the putative anti-Fusarium solani AMPs showed good quality, as indicated by the C-scores, TM-scores and RMSD values (Scholtz and Baldwin, 1992). Furthermore, the docking interaction study ascertained the binding energy displayed for HDock during detection with the putative anti- Fusarium solani AMP (OOB4) binding with the greatest affinity to Fusarium solani CUT1 protein (Zhang and Skolnick, 2004). Van der Waals forces gave the relatively weak electric forces that attracted neutral molecules between the anti-*Fusarium solani* AMPs and CUT1 protein (Hermann and Tkatchenko, 2020). The electrostatic energy referred to the electromagnetic gradient, which occurred when there were no moving electrical charges between the anti-*Fusarium solani* AMPs and *Fusarium solani* CUT1 protein (Wang et al., 2020). In the aqueous environment; desolvation energy was generated with the behavioural pattern of the CUT1 protein and anti-Fusarium solani AMPs (Hou et al., 2020). Overall, the pyDockWeb server had all the AMPs with significant values for these energy values, with OOB1 having the greatest electrostatic and desolvation energies and the lowest van der Waals interaction.

The tendency of the putative AMPs generated from this study to generate appreciable binding potential to CUT1 protein of *Fusarium solani* can be pursued for the rational design of a novel, selective and potent biomarker for the identification of *Fusarium solani*. Thus, this research produces new insights into the *in silico* modular architecture of the evolutionarily conserved host defense molecules such as AMPs with diagnostic helices associated with antimicrobial activity against fungal pathogens such as *Fusarium solani*.

## Conclusion

The present research used *in silico* analysis to detect biomolecules for the sensitive identification of *Fusarium solani*. AMPs have shown great promise in circumventing the drawbacks associated with the current diagnostic systems. Five anti-*Fusarium solani* AMPs were identified with significant physicochemical parameters to detect *Fusarium solani* using CUT1 receptor protein as a target. The generated AMPs were sensitive and specific against *Fusarium solani*. OOB1 ranked the most significant binding energy interaction, while OOB1 had the most effective interaction with *Fusarium solani* CUT1 protein. The putative anti-*Fusarium solani* AMPs generated from this analysis could be used to prevent the catastrophic losses of the crops mentioned earlier due to the fungus, ranging from reducing the harvest quality and plant survivorship to aiding plant competitive ability.

## Data Availability

The datasets presented in this study can be found in online repositories. The names of the repository/repositories and accession number(s) can be found in the article/Supplementary Material.
