# Supplementary material for: In silico discovery of biomarkers for the accurate and sensitive detection of Fusarium solani
Source: Front Bioinform. 2022 Sep 30;2:972529. doi: 10.3389/fbinf.2022.972529 (PMC9580926; doi:10.3389/fbinf.2022.972529)
Supplement: Supplementary file 1 [file DataSheet1.docx]

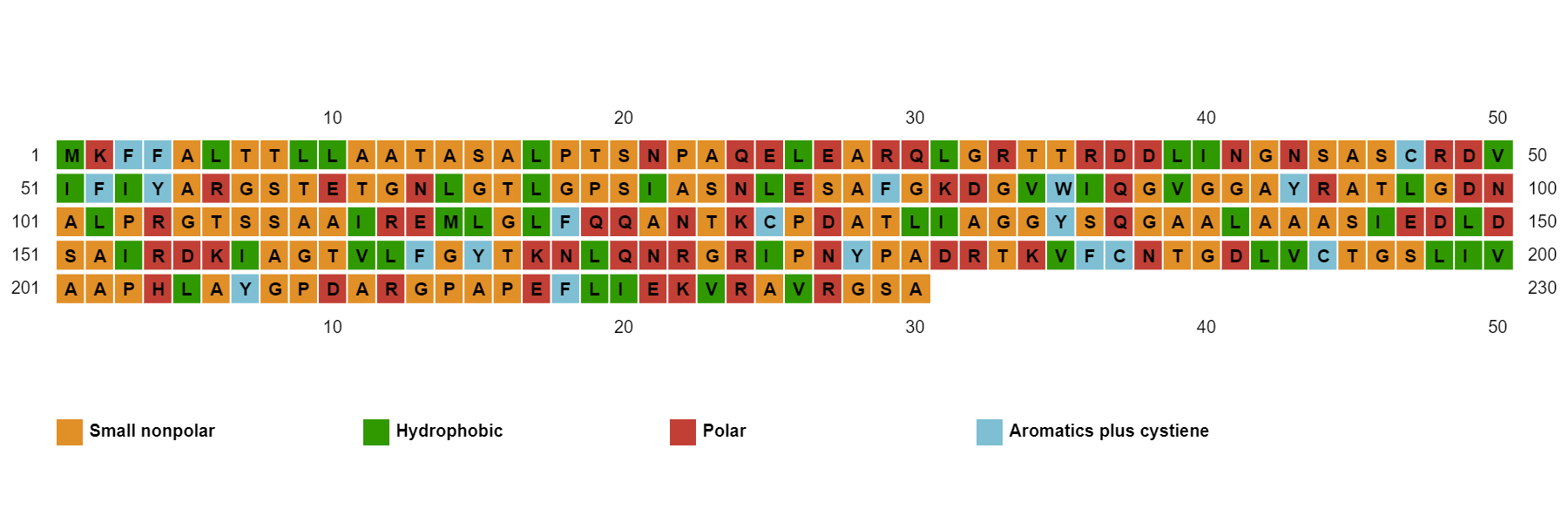


**Supplementary 1:** 2-D Secondary structure validation of the small non-polar, hydrophobic, polar, and aromatics plus cysteine regions of CUT1 protein of *Fusarium solani* using PSIPRED.


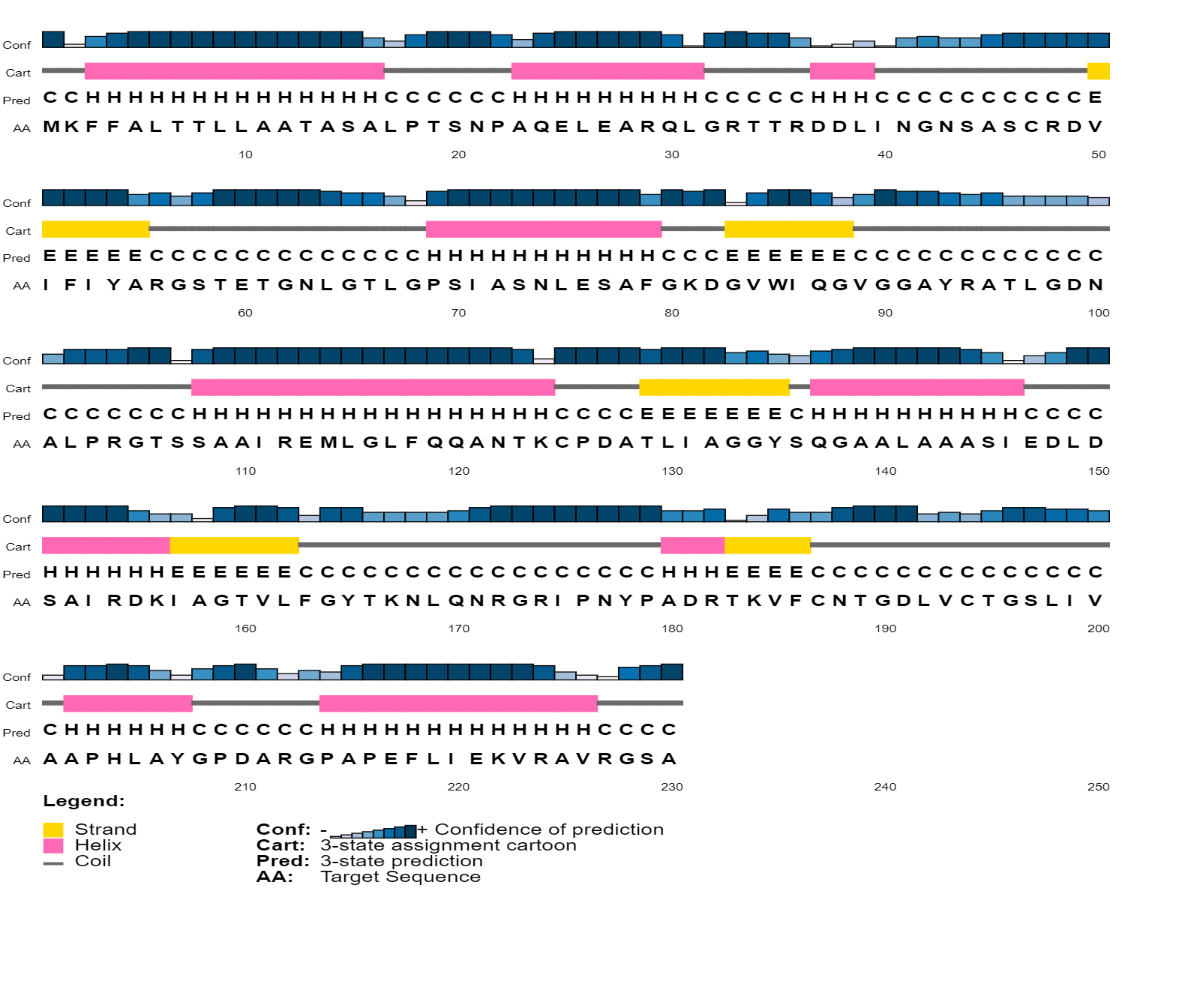


**Supplementary 2:** Secondary structure prediction of alpha-helices, beta-sheets, and random coils of *Fusarium solani* CUT1 protein using PSIPRED.


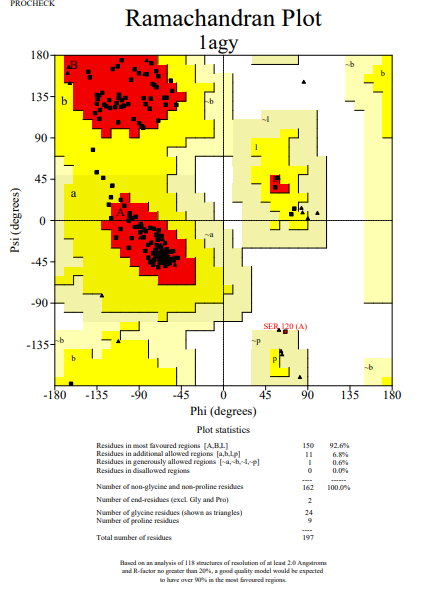


**Supplementary 3:** CUT1 protein modelled from PROCHECK using the generated model from I-TASSER. Residues in most favoured regions (A, B, L), Residues in additional allowed regions (a, b, l, p) and residues in generously allowed regions (~a, ~b ~l, ~p).
